# Toxoplasma gondii seropositivity in patients with depressive and anxiety disorders

**DOI:** 10.1016/j.bbih.2020.100197

**Published:** 2020-12-31

**Authors:** Nienke J. de Bles, Juliette E.H. van der Does, Laetitia M. Kortbeek, Agnetha Hofhuis, Gerard van Grootheest, Albert M. Vollaard, Robert A. Schoevers, Albert M. van Hemert, Brenda W.J.H. Penninx, Nathaly Rius-Ottenheim, Erik J. Giltay

**Affiliations:** a Department of Psychiatry, Leiden University Medical Center, Leiden, the Netherlands; b National Institute for Public Health and the Environment (RIVM), Centre for Disease Control the Netherlands, Bilthoven, the Netherlands; c Department of Psychiatry, Amsterdam Public Health Research Institute, Amsterdam UMC, Vrije Universiteit, Amsterdam, the Netherlands; d Department of Psychiatry, University Medical Center Groningen, Groningen, the Netherlands

**Keywords:** *Toxoplasma gondii*, Depression, Anxiety, Cognitive reactivity, Suicidality

## Abstract

**Introduction:**

*Toxoplasma gondii (T. gondii)* is an obligate intracellular parasite that is estimated to be carried by one-third of the world population. Latent *T. gondii* infection has been linked to several neuropsychiatric mood disorders and behaviors. The aim of the present study was to examine whether *T. gondii* seropositivity is associated with affective disorders, as well as with aggression reactivity and suicidal thoughts.

**Methods:**

In the Netherlands Study of Depression and Anxiety (NESDA), *T. gondii* antibodies were assessed in patients with current depressive (n ​= ​133), anxiety (n ​= ​188), comorbid depressive and anxiety (n ​= ​148), and remitted disorders (n ​= ​889), as well as in healthy controls (n ​= ​373) based on DSM-IV criteria. Seropositivity was analyzed in relation to disorder status, aggression reactivity and suicidal thoughts using multivariate analyses of covariance and regression analyses.

**Results:**

Participants were on average 51.2 years (*SD* ​= ​13.2), and 64.4% were female. Seropositivity was found in 673 participants (38.9%). A strong positive association between *T. gondii* seropositivity and age was observed. No significant associations were found between *T. gondii* seropositivity and disorder status, aggression reactivity and suicidal thoughts. The adjusted odds ratio (OR) for any remitted disorder versus controls was 1.13 (95% CI: 0.87-1.49), and for any current disorder versus controls was 0.94 (95% CI: 0.69-1.28).

**Conclusions:**

No evidence was found for a relationship between affective disorders and *T. gondii* infection in the current sample.

## Highlights

• Increasing age is strongly associated with a higher seroprevalence of *T. gondii*.
• No relationship was found between affective disorders and *T. gondii* infection.
• Affective symptom severity measures were not associated with *T. gondii* infection.
• Aggressive and suicidal thoughts were not associated with *T. gondii* infection.

## 1 Introduction

*Toxoplasma gondii* (*T. gondii*) is an obligate intracellular parasite estimated to be carried by one-third of the world’s population, making it one of the most successful human parasites (Halonen and Weiss, 2013; Montoya and Liesenfeld, 2004). It can infect mammals and many other warm-blooded animals, with cats and other felidae as its definitive hosts for sexual reproduction and the only mammals known to shed *T. gondii* oocysts with their faeces (Miller et al., 2009). In the intermediate host, the parasite can lead to a lifelong, latent infection in various tissues, including muscles, the eye and the central nervous system, where it forms persistent cysts. Humans are infected by swallowing *T. gondii* tissue cysts in contaminated food (undercooked infected meat) or oocysts in water, or through environmental exposure (e.g. through gardening).

While an acute infection is commonly asymptomatic or presenting with nonspecific symptoms like fatigue or lymphadenopathy, it can in some cases lead to toxoplasmic encephalitis. In case of a congenital toxoplasmosis, most lesions will be seen in the eyes and in the brain (e.g., intracranial calcifications, hydrocephalus). The parasite will not be cleared and, after an acute symptomatic phase, the parasite will be present as a latent infection that is commonly thought to be asymptomatic (Saadatnia and Golkar, 2012). However, there are indications of more subtle behavioral or psychological consequences. Evidence is also accumulating for an association between *T. gondii* infection and schizophrenia (Fuglewicz et al., 2017). Additionally, an increase in *T. gondii* immunoglobulin G (IgG) titers, but not in immunoglobulin M (IgM), was found among patients with schizophrenia (Ibrahim Ali et al., 2020; Leweke et al., 2004). This may implicate a latent *T. gondii* infection rather than an acute infection where both IgG and IgM titers would have increased. Increased IgG titers are thought to represent reactivation with release of tachyzoites, which may lead to psychopathology (Hester et al., 2012). The interest in the possible link between *T. gondii* and depression was sparked by a case report (Kar and Misra, 2004), describing a depressed patient who possibly remitted after antibiotic treatment of his latent *T. gondii* infection. Subsequent studies investigated the possible link between *T. gondii* infection and depression, yet, provided no clear consensus on this association. Several studies found positive associations between depressive symptoms and *T. gondii* seropositivity (Bak et al., 2018; Bay-Richter et al., 2019; Duffy, 2015). Additionally, two studies found a dose-response relationship, with higher IgG titers associated with higher depressive symptoms (Groer et al., 2011; Suvisaari et al., 2017). However, a negative association between diagnoses of depression and *T. gondii* seropositivity in women was observed in a cross-sectional study of 1486 subjects (Flegr and Escudero, 2016). Taking both genders together in one analysis, however, the association with *T. gondii* seropositivity did not longer persist. In concordance, a systematic review including ten studies with data on major depressive disorder (MDD), found no support for a significant association between *T. gondii* infection and depression, with an odds ratio (OR) of only 1.21 (95% confidence interval [CI]: 0.86-1.70) (Sutterland et al., 2015), which is in line with recent cross-sectional studies on MDD and *T. gondii* infection (Gale et al., 2014; Markovitz et al., 2015; Sugden, 2016).

Besides depression, anxiety disorders have also been studied in relation to *T. gondii* infection. An association between *T. gondii* seropositivity and generalized anxiety disorder (GAD) has been reported, with ORs ranging from 2.05 to 2.25 (Bay-Richter et al., 2019; Markovitz et al., 2015; Suvisaari et al., 2017), with an even higher OR of 4.17 among children (Akaltun et al., 2018). On the other hand, a large population-based cross-sectional study of 1846 participants (Gale et al., 2014) reported no significant associations between *T. gondii* infection and GAD or panic disorder (PD).

Furthermore, latent *T. gondii* infection has been linked to behavioral changes such as increased feelings of aggression and anger (Coccaro, 2016; Cook, 2015; Duffy, 2015) and self-directed violence (e.g., suicide attempts, completed suicide, and self-directed violence) (Alvarado-Esquivel et al., 2013; Arling et al., 2009; Bak et al., 2018; Okusaga et al., 2011; Pedersen et al., 2012; Sutterland et al., 2019). A study of 1000 participants without any psychiatric diagnoses showed an association between *T. gondii* seropositivity and trait reactive aggression among women (Cook, 2015), which was in line with a smaller study among 70 female veterans showing a relationship between *T. gondii* seropositivity and higher anger scores (Duffy, 2015). Another previous study on *T. gondii* seropositivity and anger included both healthy participants and psychiatric patients. This cross-sectional study of 358 adults found an association between *T. gondii* seropositivity and higher aggression and impulsivity. Additionally, seropositivity was highest in subjects with intermittent explosive disorder (21.8% of n ​= ​110), compared to healthy controls (9.1% of n ​= ​110), and other psychiatric patients (16.7% of n ​= ​138) (Coccaro, 2016). Several studies on self-harm have also been conducted. Cross-sectional and case-control studies focusing on suicide attempts and *T. gondii* infection found evidence for a relationship with seropositivity, with ORs ranging from 1.57 to 7.12 (Bak et al., 2018; Okusaga et al., 2011; Zhang et al., 2012), and antibody titers, meaning that higher IgG titers were associated with suicide attempts (Alvarado-Esquivel et al., 2013; Arling et al., 2009; Okusaga et al., 2011; Zhang et al., 2012). In a large register-based prospective cohort study among 45,788 women after child birth, *T. gondii*–infected mothers had a relative risk of self-directed violence of 1.53 (95% CI, 1.27-1.85) versus non-infected counterparts (Pedersen et al., 2012). Thus, there seems to be an association between *T. gondii* latent infection on the one hand and (self-directed) aggression and suicidality on the other hand.

We aimed to further examine the link between *T. gondii* infection and (comorbid) depressive and anxiety disorders in a large cohort study. Based on existing literature, we hypothesized [1] that *T. gondii* seropositivity is associated with the presence and severity of depressive and anxiety disorders, and [2] that *T. gondii* seropositivity is associated with aggression reactivity and suicidal thoughts.

## 2 Methods

### 2.1 Participants

Participants were selected from the Netherlands Study of Depression and Anxiety (NESDA), an ongoing multisite, naturalistic, longitudinal cohort study. NESDA was designed to examine the long-term course and consequences of depressive and anxiety disorders. A total of 2981 participants (18–65 years) were enrolled at baseline. This population was composed of participants with current or remitted depressive and anxiety disorders, and comorbid depressive and anxiety disorders. The control group consisted of participants without lifetime psychiatric disorders. Participants were recruited from the community, primary care, and specialized (mental) health care in the vicinity of the clinical sites (i.e., Amsterdam [Northwest], Leiden [Southwest], Groningen [Northeast]). Exclusion criteria were (1) the presence of other psychiatric disorders (e.g., psychotic, obsessive–compulsive, bipolar, or severe addiction disorder): and (2) not being fluent in Dutch. All participants gave written informed consent before enrolment, and the ethical committees of participating universities (VU University Medical Center, Leiden University Medical Center, and University Medical Center Groningen) granted ethical approval. A more detailed description of NESDA is given elsewhere (Penninx et al., 2008).

For the current cross-sectional study, data was gathered at the 6th wave at 9-year follow-up between 2014 and 2017. Participants who completed the 6th wave totaled 2069 (69.4%), of whom 1731 titers were obtained and used for the current analyses. Excluded participants were younger (*p* ​= ​0.004), more often women (*p* ​< ​0.001) and had more fear symptoms (*p* ​< ​0.001) compared to the sample included in the current study.

### 2.2 Measurements

#### 2.2.1 Toxoplasma gondii IgG antibodies

Citrated plasma samples were kept frozen at −80 ​°C until essayed for *T. gondii* IgG antibodies. In response to the parasite, *T. gondii* IgG antibodies are produced within the first two to three weeks after infection, peaks at 3 months and, although it can decrease slowly, remains detectable over the individual’s lifetime. IgG antibody levels were assayed in duplicate in plasma using a sandwich Enzyme Linked Immunosorbent Assay (ELISA) with a plasma dilution of 1:20 (adapted from a previously described method (Ruitenberg and van Knapen, 1977)). Detection of IgG antibodies in citrated plasma was first validated in a subpopulation of 100 participants, by comparing *T. gondii* seropositivity in citrated plasma from the 6th wave with serum samples from the 5th wave. The agreement for IgG antibodies in serum and citrated plasma was 100% (Suppl. Fig. 1). The time span between blood collection at these waves ranged from 28 to 56 months. The sensitivity and specificity of the ELISA were 99–100% and 90–99% respectively. The antigen is derived from a crude extract of a *Toxoplasma* RH strain, the conjugate is a peroxidase-labelled anti-human IgG conjugate (Dako, Denmark). A cut-off serum was used and its optical density (OD) value was allowed to vary between 0.10 and 0.30. The extinction value of the tested sample and the cut-off serum was used to calculate a ratio. A subject with a ratio of at least 1.0 was considered to be seropositive for *T. gondii* (Hofhuis, 2011).

### 2.3 Depressive and anxiety disorders

Diagnoses of depression (i.e., MDD, dysthymia) and anxiety disorders (i.e., social phobia [SP], PD, GAD, agoraphobia [AP]) were established with the Composite International Diagnostic Interview (CIDI; WHO version 2.1). The CIDI is a fully structured clinical interview based on criteria of the fourth edition of the Diagnostic and Statistical Manual of Mental Disorders (DSM-IV (APA, 1994)). Based on the CIDI information, all 1731 NESDA participants were divided into the following psychopathology groups: [1] healthy participants who have no current and past history of psychiatric disorders (n ​= ​373); [2] participants with a lifetime history of a depressive or anxiety disorder, but not in the last 6 months (n ​= ​889); [3] patients with a current depressive (n ​= ​133) or [4] anxiety disorder (n ​= ​188); and [5] patients with current comorbid depressive and anxiety disorders (n ​= ​148). The CIDI was established at the same day of blood collection.

### 2.4 Symptom severity

The severity of depressive symptoms was assessed with the Inventory of Depressive Symptomatology (self-report version; IDS-SR) (Rush et al., 1986; Rush et al., 1996). The IDS-SR is a 30-item questionnaire scored on a 4-point Likert scale (0–3), with sum scores ranging from 0 to 84 (28 out of 30 items are rated). The internal consistency (i.e., Cronbach’s alpha) of the IDS-SR in our sample was 0.89. The Beck Anxiety Inventory (BAI) is a 21-item self-report questionnaire for measuring the somatic symptoms of anxiety (Beck et al., 1988). It uses a 4-point Likert scale (0–3), with total scores ranging from 0 to 63. The current sample showed an internal consistency (i.e., Cronbach’s alpha) of 0.92. Additionally, the 15-item self-rating Fear Questionnaire (FQ) was obtained to measure phobias and, particularly, related avoidance (Marks and Mathews, 1979), on a 9-point Likert scale (0–8), with total scores ranging from 0 to 120 and a Cronbach’s alpha of 0.89 in the current sample. The abbreviated 11-item version of the Penn State Worry Questionnaire (PSWQ) was assessed to measure pathological worry and general anxiety (Meyer et al., 1990). Items are scored on a 5-point Likert scale (1–5), with sum score ranging from 11 to 55. Our sample presented an internal consistency (i.e., Cronbach’s alpha) of 0.96.

### 2.5 Cognitive reactivity

The revised Leiden Index of Depression Sensitivity (LEIDS-R) is a 34-item self-report questionnaire developed to measure cognitive reactivity to sad mood (Van der Does, 2002; Van der Does and Williams, 2003). Items are divided into six reactivity subscales, of which the subscales aggression and hopelessness/suicidality were used in the current wave. Items are filled out on a 5-point Likert scale ranging from 0 to 4. The aggression subscale constitutes of 6 items (e.g. ‘In a sad mood, I am bothered more by aggressive thoughts’), with a maximum score of 24. The hopelessness/suicidality subscale constitutes of 5 items (e.g. ‘When I feel sad, more thoughts of dying or harming myself go through my mind’), with a maximum score of 20. The internal consistency (i.e., Cronbach’s alpha) of the LEIDS-R subscales in our sample were 0.83 and 0.87, respectively.

### 2.6 Covariates

Sociodemographic covariates consisted of sex, age, education (in years), North European ancestry (yes/no), and clinical site location (i.e., Amsterdam, Leiden, Groningen). Age was divided into four age groups (i.e., 40 and younger; 41–50; 51–60; 61 and older). Body Mass Index (BMI) was calculated based on measured weight and height.

### 2.7 Statistical analyses

Sociodemographic characteristics were described within the *T. gondii* seronegative and seropositive groups using chi-squared tests for categorical variables and t-tests (ANOVA) for continuous variables. We also performed multivariable logistic regression analyses to examine the associations of *T. gondii* seropositivity according to demographic characteristics.

Chi-squared tests for independent samples were conducted to compare the prevalence of seropositivity among psychiatric disorders. Using multivariable logistic regression, these comparisons were repeated, adjusting for sex, age, level of education, North European ancestry, BMI, and clinical site location. Subsequently, we adjusted the full model according to previous mentioned sociodemographic variables and healthy controls, remitted depression and/or anxiety, and current dysthymia, MDD, SP, PD, AP, and GAD. A forest plot was used to examine the OR (with 95% CI) of seropositivity among psychiatric diagnoses. In addition to testing for dichotomous seropositivity, sensitivity analyses were performed using multivariable linear regression for the continuous level of *T. gondii* IgG antibodies expressed as the ratio of OD values. The level of T. gondii IgG antibodies was naturally log transformed in order for its distribution to approach normality.

We also performed t-tests for independent samples to examine the association of symptom severity measures and cognitive reactivity (i.e., aggression and suicidality) with seropositivity. Using analysis of covariance (ANCOVA), analyses were repeated adjusting for sex, age, level of education, North European ancestry, BMI, and clinical site location. A second forest plot was used to examine the association of seropositivity with symptom severity and cognitive reactivity. Subsequently, sensitivity analyses were performed for the transformed level of *T. gondii* IgG antibodies in multivariable linear regression. A two-tailed significance level of *p* ​< ​0.05 was considered statistically significant for all analyses. The Benjamini-Hochberg (B–H) correction was performed in order to correct for a false discovery rate (FDR) in multiple comparisons (Benjamini and Hochberg, 1995). Analyses were performed using IBM SPSS statistical software (version 25, IBM Corp.).

## 3 Results

**Fig. 1:**
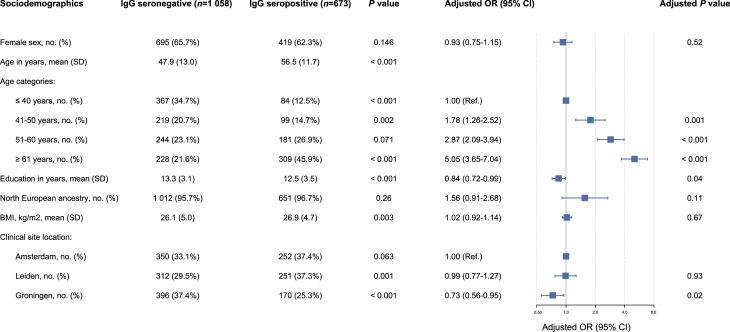
Characteristics of the study sample (*N* ​= ​1731) according *T. gondii* seropositivity and the adjusted odds ratios for all independent variables in one multivariable logistic regression model. *Note.* BMI = Body Mass Index; Chi-square values have been computed for categorical variables, ANOVA for interval variables; Education in years and BMI were studied per 5 units (i.e., 5 ​kg/m^2^) increase.

The mean age of the participants (*N* ​= ​1731) was 51.2 years (*SD* ​= ​13.2), and 64.4% were female. As shown in Fig. 1, 673 participants (38.9%) were seropositive for *T. gondii* antibodies. The odds of being seropositive for *T. gondii* increased strongly with age. *T. gondii* seroprevalence differed per clinical site location, ranging from 30.0% in Groningen (Northeast), 41.9% in Amsterdam (Northwest), to 44.6% in Leiden (Southwest). The area remained independently associated with *T. gondii* infection after adjustment for demographic characteristics. However, this was only true for Groningen compared to Amsterdam and Leiden, but not for Amsterdam and Leiden compared to each other.

**Fig. 2:**
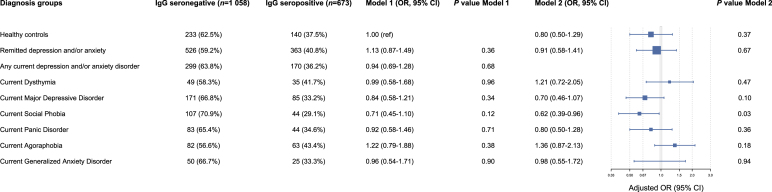
The (adjusted) odds ratios of seropositivity for depressive and anxiety disorders. Model 1 adjusted for sex, age, level of education, North European ancestry, BMI, and clinical site location with healthy controls taken as the reference group. Model 2 adjusted for the beforementioned sociodemographic variables, healthy controls, remitted depression and/or anxiety, and current dysthymia, MDD, SP, PD, AP, and GAD.

Fig. 2 shows the adjusted odds of being seropositive for depressive and anxiety disorders. Healthy controls were taken as the reference group in Model 1. No associations were found between seropositivity and depressive and anxiety diagnoses after adjusting for sociodemographic variables. The odds ratio (OR) for any remitted disorder versus controls was 1.13 (95% CI: 0.87-1.49), and for any current disorder versus controls was 0.94 (95% CI: 0.69-1.28). The fully adjusted model only showed a significant negative association between *T. gondii* seropositivity and SP (OR ​= ​0.62; 95% CI: 0.39-0.96). However, after B–H correction, the result was no longer deemed statistically significant. When these analyses were repeated for *T. gondii* IgG antibodies, no significant associations were found in the models adjusting for sociodemographic variables. The fully adjusted model resulted in a significant negative association with *T. gondii* and MDD (*β* ​= ​−0.09; *p* ​= ​0.008), and with *T. gondii* and SP (*β* ​= ​−0.06; *p* ​= ​0.03; Suppl. Table 2). Again, these associations were no longer deemed statistically significant after B–H correction.

**Fig. 3:**
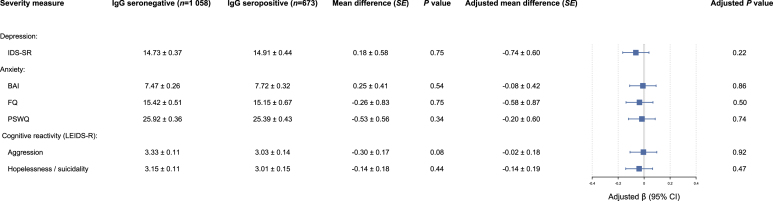
Mean differences (with standard errors in parentheses) between IgG seronegative and seropositive subjects on symptom severity measures (i.e., IDS-SR, BAI, FQ, and PSWQ), aggression reactivity and suicidal thoughts. Adjusted for sex, age, level of education, North European ancestry, BMI, and clinical site location. *Note:* IDS-SR = Inventory of Depressive Symptomatology – self-report; BAI = Beck Anxiety Inventory; FQ = Fear Questionnaire; PSWQ = Penn State Worry Questionnaire; LEIDS-R ​= ​Leiden Index of Depression Sensitivity – Revised.

As shown in Fig. 3, no mean differences were found between seropositive and seronegative subjects on symptom severity measures (i.e., IDS-SR, BAI, FQ, and PSWQ), aggression reactivity and suicidal thoughts. These associations remained non-significant after adjustment for sociodemographic variables. All severity measures were also tested for seropositivity in diagnostic strata (i.e., controls, remitted, and current psychopathology), in which no important differences in effect sizes among the groups were found, and none of the comparisons were statistically significant (data not shown). Analyses for a crude and adjusted model were repeated for antibody levels and also found no significant relationships (Suppl. Table 2).

## 4 Discussion

This study aimed to examine the link between *T. gondii* specific IgG antibodies and disorder status, aggression reactivity and suicidal thoughts. No significant association was found for *T. gondii* seropositivity in relation to disorder status. Similarly, no significant associations were found for *T. gondii* seropositivity in relation to aggression reactivity or suicidal thoughts. *T. gondii* seropositivity was strongly associated with older age.

Our findings of a lack of the association between *T. gondii* seropositivity and depression diagnosis is in line with the before-mentioned meta-analysis (Sutterland et al., 2015) and several population-based cross-sectional studies (Gale et al., 2014, Markovitz et al., 2015; Sugden, 2016). We extended their findings by showing that there was no association neither with self-reported depressive symptoms nor with observer-rated depression diagnoses through standardized diagnostic psychiatric interviews. Although, some significant associations between seroprevalence and depressive symptoms were found in some studies, these findings were done in studies with smaller sample sizes of at most 51 seropositive subjects, increasing the risk of chance findings (Bak et al., 2018; Bay-Richter et al., 2019; Duffy, 2015). A dose-response relationship, with higher antibody titers being associated with an increase in depressive symptoms, was reported in two studies (Groer et al., 2011; Suvisaari et al., 2017). The majority of studies, however, did not find any associations (Bay-Richter et al., 2019, Gale et al., 2014; Markovitz et al., 2015), which was in line with our findings. Previous inconsistencies could be explained neither by differences in age distribution nor by the strain hypothesis which states that strains of *T. gondii* differ in virulence and in ability to influence human behavior (Abdoli, 2013; Xiao et al., 2009; Xiao et al., 2011). Hence, the current study bolsters the rejection of the hypothesis that *T. gondii* seropositivity is associated with the presence and severity of major depression.

In line with our findings on depressive status, we did not find associations between *T. gondii* seropositivity and anxiety disorders. This was concordant with population-based studies of 1846 and 7712 participants respectively that did not find an association between *T. gondii* and GAD or PD (Gale et al., 2014), and between *T. gondii* and PD, AP or SP (Suvisaari et al., 2017), as established with the CIDI. The latter study only found a significant relationship between *T. gondii* seropositivity and GAD, yet no corrections for multiple comparisons were used (Suvisaari et al., 2017). Two other large population-based studies reported a significant relationship between *T. gondii* seropositivity and GAD as established with a telephone survey (Markovitz et al., 2015), and general anxiety based on a screening tool (Bay-Richter et al., 2019). These results contradict animal studies that suggest reduced anxiety-like behavior in *T. gondii* infected rodents (Berdoy, 2000; Vyas et al., 2007).

The association of seropositivity with aggression reactivity was inconsistent with previously reported studies on self-reported anger and aggressive behavior (Coccaro, 2016; Cook, 2015; Duffy, 2015). However, those studies used other but related constructs (i.e., anger mood, aggressive tendencies as a personality trait, and a history of actual aggressive behavior), although one study also measured aggression reactivity (Cook, 2015). The latter study found significant results only among women. Importantly, aggressive reactivity measures thoughts rather than actual behavior like the history of aggression. The current study also took into account self-directed aggression by measuring suicidal thoughts. No differences were found between seropositive and seronegative subjects, which contradicts the previous studies that found an association between *T. gondii* infection and suicidality (Alvarado-Esquivel et al., 2013; Arling et al., 2009; Bak et al., 2018; Okusaga et al., 2011; Pedersen et al., 2012; Zhang et al., 2012). However, some of these studies reported inconsistent findings, with a significant relationship of suicide attempts with seropositivity but not with antibody levels (Bak et al., 2018), or the inverse (Alvarado-Esquivel et al., 2013; Arling et al., 2009). Furthermore, the significant associations that were reported in Okusaga et al. (2011) were only found among patients under 38, while no association for seropositivity or antibody levels was found among older patients. In addition, as most of these studies had a cross-sectional design, there remains the possibility of reverse causation, meaning that disorder status or behavioral traits may have affected the risk of *T. gondii* infection (Cook, 2015; Markovitz et al., 2015; Sutterland et al., 2019).

The seroprevalence of *T. gondii* strongly increased with age, which is a result of cumulative seropositivity and is in line with previous studies (Hofhuis, 2011; Kortbeek et al., 2004; Montoya and Liesenfeld, 2004). Although some studies found differences between males and females (Cook, 2015; Flegr and Escudero, 2016), others, including ours, did not (Markovitz et al., 2015). Our finding that seroprevalence was independently associated with geographical regions was concordant with previous results from the Netherlands (Hofhuis, 2011). These previous results indicated highest seroprevalence rates in the Northwest (43%) and Southwest (37%) regions, compared to other provinces in the Netherlands. They also found a steepest rise in seroprevalence in the age group 15–49 years. Importantly, the current sample had a mean age of 51.2 years. Furthermore, a substantial part of the sample was recruited in Western regions. These two factors may explain our relatively high seroprevalence rate of 38.9%.

This study has several strengths. We investigated seroprevalence in a large cohort that included patients without lifetime psychiatric disorders (“control subjects”), with (current and remitted) depressive and anxiety disorders, or comorbid depressive and anxiety disorders. Diagnoses were established with the CIDI (WHO version 2.1), a comprehensive observer-rated instrument with high interrater reliability (Wittchen et al., 1991), high test–retest reliability (Wacker et al., 1990) and high validity for depressive and anxiety disorders (Wittchen, 1994; Wittchen et al., 1989). Moreover, we not only stratified participants according to their diagnosis, but also studied symptom severity levels. Furthermore, blood samples were assayed according to a reliable standardized in-house ELISA protocol of the RIVM, with a sensitivity and specificity of 99–100% and 90–99% respectively. The methods, antigens and controls have not altered over the past 35 years, making the results of the different studies comparable.

Limitations of the current study must also be mentioned. The cross-sectional design of the current study hampers inferences of causation, therefore prospective studies are still needed. A second limitation is that the difference between strains (i.e., Types I, II, III, and atypical or recombinant strains) which causes chronic infection is not accounted for. Since we assume that infections with distinct strains all result in increased titers, seropositivity itself cannot be used to differentiate between strains or help to unravel differences in (neuro)virulence between strains. We also did not exclude other infections and immune system diseases. Some studies suggest that *T. gondii* antibodies may only be an indicator of previous contacts with cats, with these cats carrying other pathogens such as *Bartonella henselae* affecting mental health (Flegr and Hodny, 2016; Flegr et al., 2018; Yuksel, 2010). A last limitation is that we did not study actual suicidal and aggressive behaviors.

In conclusion, the current study does not support the hypothesis that *T. gondii* seropositivity is associated with depressive or anxiety disorders, or with aggressive and suicidal thoughts. In light of previous studies and our new findings, it seems unlikely that *T. gondii* seropositivity plays a major role in the risk of affective disorders, suicidality and aggressive thoughts.

## Data availability statement

An a priori analysis plan for this study was approved by the principal investigator of NESDA and the NESDA board. Because of ethical and legal restrictions, data involving clinical participants are not included in the manuscript or made available in a public repository. However, subject to approval, data are available upon request from the NESDA Data Access Committee (nesda@ggzingeest.nl).

## Declaration of competing interest

The authors declare that they have no known competing financial interests or personal relationships that could have appeared to influence the work reported in this paper.
