# Supplementary figures and images for: Toxoplasma gondii seropositivity in patients with depressive and anxiety disorders
Source: Brain Behav Immun Health. 2020 Dec 31;11:100197. doi: 10.1016/j.bbih.2020.100197 (PMC8474384; doi:10.1016/j.bbih.2020.100197)

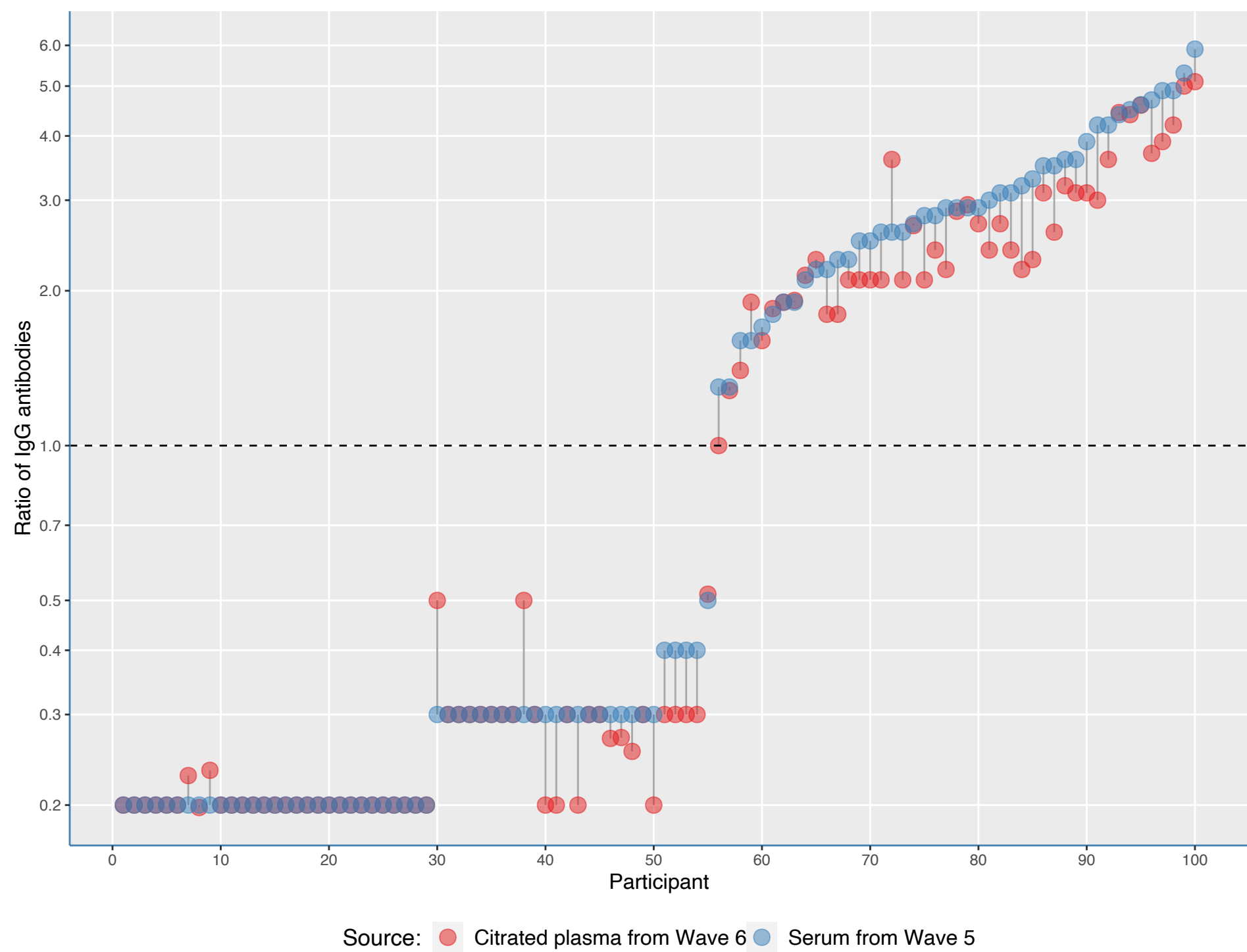

Supplement: Multimedia component 1 [file mmc1.pdf]
