# Supplementary material for: Toxoplasma gondii seropositivity in patients with depressive and anxiety disorders
Source: Brain Behav Immun Health. 2020 Dec 31;11:100197. doi: 10.1016/j.bbih.2020.100197 (PMC8474384; doi:10.1016/j.bbih.2020.100197)
Supplement: Multimedia component 2 [file mmc2.docx]

**De Bles et al., *T. gondii* in depression and anxiety**

Supplementary Table 1. *T. gondii* IgG antibody levels according to diagnosis groups.

| **Diagnosis groups** | **Model 1^†^** | | **Model 2^±^** | |
| --- | --- | --- | --- | --- |
|  | ***β*** | ***P* value** | ***β*** | ***P* value** |
| Healthy controls | 1.00 (ref) |  | -0.06 | 0.15 |
| Remitted depression and/or anxiety | 0.03 | 0.50 | -0.06 | 0.25 |
| Any current depression and/or anxiety disorder | 0.09 | 0.11 |  |  |
| Current Dysthymia | 0.03 | 0.76 | 0.02 | 0.38 |
| Current Major Depressive Disorder | -0.008 | 0.91 | -0.09 | 0.008 |
| Current Social Phobia | 0.05 | 0.54 | -0.06 | 0.03 |
| Current Panic Disorder | 0.12 | 0.13 | -0.01 | 0.58 |
| Current Agoraphobia | 0.08 | 0.30 | 0.03 | 0.36 |
| Current Generalized Anxiety Disorder | 0.05 | 0.52 | -0.004 | 0.87 |

^†^ Adjusted for sex, age, level of education, North European ancestry, BMI, and clinical site location.

^±^ Adjusted for the beforementioned sociodemographic variables, healthy controls, remitted depression and/or anxiety, and current dysthymia, MDD, SP, PD, AP, and GAD.

Supplementary Table 2. *T. gondii* IgG antibody levels according to severity measures.

| **Severity measure** | **Crude** | |  | **Adjusted Model^†^** | |
| --- | --- | --- | --- | --- | --- |
|  | ***β*** | ***P* value** |  | ***β*** | **Adjusted *P* value** |
| Depression: |  |  |  |  |  |
| IDS-SR | 0.002 | 0.93 |  | -0.03 | 0.21 |
| Anxiety: |  |  |  |  |  |
| BAI | 0.02 | 0.40 |  | 0.004 | 0.88 |
| FQ | -0.008 | 0.74 |  | -0.02 | 0.47 |
| PSWQ | -0.03 | 0.24 |  | -0.02 | 0.49 |
| Cognitive reactivity (LEIDS-R): |  |  |  |  |  |
| Aggression | -0.04 | 0.08 |  | -0.01 | 0.75 |
| Hopelessness / suicidality | -0.01 | 0.63 |  | -0.01 | 0.65 |

**^†^** Adjusted for sex, age, level of education, North European ancestry, BMI, and clinical site location.
